# Supplementary material for: Vitamin D and Periodontitis: A Systematic Review and Meta-Analysis
Source: Nutrients. 2020 Jul 22;12(8):2177. doi: 10.3390/nu12082177 (PMC7468917; doi:10.3390/nu12082177)
Supplement: Supplementary file 1 [file nutrients-12-02177-s001.pdf]

Supplementary Information

Summary

Appendix S1. PRISMA 2009 Checklist *2*

Appendix S2. List of potentially relevant studies not included in the systematic review, along with the reasons for exclusion. *4*

Appendix S3. Newcastle-Ottawa Scale *13*

Appendix S4. RoB2 Tool *13*

Appendix S5. ROBINS-I Tool *13*

Apendix S6. Summary of estimates of meta-regression to assess the influence of smoking on 25(OH)D serum levels *13*

Appendix S1. PRISMA 2009 Checklist

Section/topic	#	Checklist item	Reported on page #
TITLE			
Title	1	Identify the report as a systematic review, meta-analysis, or both.	1
ABSTRACT			
Structured summary	2	Provide a structured summary including, as applicable: background; objectives; data sources; study eligibility criteria, participants, and interventions; study appraisal and synthesis methods; results; limitations; conclusions and implications of key findings; systematic review registration number.	2
INTRODUCTION			
Rationale	3	Describe the rationale for the review in the context of what is already known.	3
Objectives	4	Provide an explicit statement of questions being addressed with reference to participants, interventions, comparisons, outcomes, and study design (PICOS).	3
METHODS			
Protocol and registration	5	Indicate if a review protocol exists, if and where it can be accessed (e.g., Web address), and, if available, provide registration information including registration number.	3-4
Eligibility criteria	6	Specify study characteristics (e.g., PICOS, length of follow-up) and report characteristics (e.g., years considered, language, publication status) used as criteria for eligibility, giving rationale.	4
Information sources	7	Describe all information sources (e.g., databases with dates of coverage, contact with study authors to identify additional studies) in the search and date last searched.	4
Search	8	Present full electronic search strategy for at least one database, including any limits used, such that it could be repeated.	4
Study selection	9	State the process for selecting studies (i.e., screening, eligibility, included in systematic review, and, if applicable, included in the meta-analysis).	4-5
Data collection process	10	Describe method of data extraction from reports (e.g., piloted forms, independently, in duplicate) and any processes for obtaining and confirming data from investigators.	5
Data items	11	List and define all variables for which data were sought (e.g., PICOS, funding sources) and any assumptions and simplifications made.	5
Risk of bias in individual studies	12	Describe methods used for assessing risk of bias of individual studies (including specification of whether this was done at the study or outcome level), and how this information is to be used in any data synthesis.	5
Summary measures	13	State the principal summary measures (e.g., risk ratio, difference in means).	5-6
Synthesis of results	14	Describe the methods of handling data and combining results of studies, if done, including measures of consistency (e.g., I^2) for each meta-analysis.	5-6

Section/topic	#	Checklist item	Reported on page #
Risk of bias across studies	15	Specify any assessment of risk of bias that may affect the cumulative evidence (e.g., publication bias, selective reporting within studies).	NA
Additional analyses	16	Describe methods of additional analyses (e.g., sensitivity or subgroup analyses, meta-regression), if done, indicating which were pre-specified.	6
RESULTS			
Study selection	17	Give numbers of studies screened, assessed for eligibility, and included in the review, with reasons for exclusions at each stage, ideally with a flow diagram.	6
Study characteristics	18	For each study, present characteristics for which data were extracted (e.g., study size, PICOS, follow-up period) and provide the citations.	6
Risk of bias within studies	19	Present data on risk of bias of each study and, if available, any outcome level assessment (see item 12).	6
Results of individual studies	20	For all outcomes considered (benefits or harms), present, for each study: (a) simple summary data for each intervention group (b) effect estimates and confidence intervals, ideally with a forest plot.	6
Synthesis of results	21	Present results of each meta-analysis done, including confidence intervals and measures of consistency.	6-7
Risk of bias across studies	22	Present results of any assessment of risk of bias across studies (see Item 15).	NA
Additional analysis	23	Give results of additional analyses, if done (e.g., sensitivity or subgroup analyses, meta-regression [see Item 16]).	7
DISCUSSION			
Summary of evidence	24	Summarize the main findings including the strength of evidence for each main outcome; consider their relevance to key groups (e.g., healthcare providers, users, and policy makers).	7
Limitations	25	Discuss limitations at study and outcome level (e.g., risk of bias), and at review-level (e.g., incomplete retrieval of identified research, reporting bias).	7-9
Conclusions	26	Provide a general interpretation of the results in the context of other evidence, and implications for future research.	9
FUNDING			
Funding	27	Describe sources of funding for the systematic review and other support (e.g., supply of data); role of funders for the systematic review.	9

NA – Not applicable

From: Moher D, Liberati A, Tetzlaff J, Altman DG, The PRISMA Group (2009). Preferred Reporting Items for Systematic Reviews and Meta-Analyses: The PRISMA Statement. PLoS Med 6(7): e1000097. doi:10.1371/journal.pmed1000097

For more information, visit: www.prisma-statement.org.

Appendix S2. List of potentially relevant studies not included in the systematic review, along with the reasons for exclusion.

Number	Reference	Reason for exclusion
1	Taskan MM, Gevrek F. PPAR-γ, RXR, VDR, and COX-2 Expressions in gingival tissue samples of healthy individuals, periodontitis and peri-implantitis patients. Niger J Clin Pract. 2020;23(1):46-53. doi:10.4103/njcp.njcp_349_19	Unrelated
2	Schütz JDS, de Azambuja CB, Cunha GR, et al. Association between severe periodontitis and chronic kidney disease severity in predialytic patients: A cross-sectional study. Oral Dis. 2020;26(2):447-456. doi:10.1111/odi.13236	No periodontitis group
3	Munhoz Pereira T, Alvim-Pereira F, Kaiser Alvim-Pereira CC, Ignácio SA, Machado de Souza C, Trevilatto PC. A complete physical mapping of the vitamin D receptor gene for dental implant loss: A pilot study. Clin Oral Implants Res. 2019;30(12):1165-1178. doi:10.1111/clr.13529	Unrelated
4	Fomenko IG, Harbuzova VY, Obukhova OA, Pohmura VV, Plakhtiienko IA, Piven SN. The association of apai-polymorphism of vitamin D receptor gene (VDR) with development of generalized parodontitis in Ukrainian population. Wiad Lek. 2019;72(7):1253-1257.	Unable to access
5	Murthykumar K, Arjunkumar R, Jayaseelan VP. Association of vitamin D receptor gene polymorphism (rs10735810) and chronic periodontitis. J Investig Clin Dent. 2019;10(4):e12440. doi:10.1111/jicd.12440	Unrelated
6	Rafique S, Hingorjo MR, Mumtaz M, Qureshi MA. The relationship of 1,25-dihydroxyvitamin D and Vitamin D binding protein in periodontitis. Pak J Med Sci. 2019;35(3):847-851. doi:10.12669/pjms.35.3.482	Incomplete data
7	Bonnet C, Rabbani R, Moffatt MEK, Kelekis-Cholakis A, Schroth RJ. The Relation Between Periodontal Disease and Vitamin D. J Can Dent Assoc. 2019;84:j4.	Unsuitable control group
8	Marian D, Rusu D, Stratul SI, Calniceanu H, Sculean A, Anghel A. Association of Vitamin D Receptor Gene Polymorphisms with Chronic Periodontitis in a Population in Western Romania. Oral Health Prev Dent. 2019;17(2):157-165. doi:10.3290/j.ohpd.a39738	Unable to access
9	Guido Mangano F, Ghertasi Oskouei S, Paz A, Mangano N, Mangano C. Low serum vitamin D and early dental implant failure: Is there a connection? A retrospective clinical study on 1740 implants placed in 885 patients. J Dent Res Dent Clin Dent Prospects. 2018;12(3):174-182. doi:10.15171/joddd.2018.027	Data presented in thresholds
10	Pinho RCM, Dias RSAM, Bandeira F, et al. Polymorphisms of the vitamin D receptor gene (FOKI, CDX2, and GATA) and susceptibility to chronic periodontitis in diabetic and non-diabetic individuals: A case-control study. J Investig Clin Dent. 2019;10(1):e12370. doi:10.1111/jicd.12370	Unrelated
11	Ratheesh V, Subramanian S, Prakash PSG, Victor DJ. Evaluation of Association of Vitamin D Receptor Genetic Polymorphism with Severe Chronic Periodontitis in an Ethnic Tamilian Population. Genet Test Mol Biomarkers. 2018;22(10):615-621. doi:10.1089/gtmb.2018.0190	Unrelated
12	Romandini M, Laforí A, Romandini P, Baima G, Cordaro M. Periodontitis and platelet count: A new potential link with	No control group

	cardiovascular and other systemic inflammatory diseases. J Clin Periodontol. 2018;45(11):1299-1310. doi:10.1111/jcpe.13004	
13	Nazemisalman B, Vahabi S, Sabouri E, Hosseinpour S, Doaju S. Association of vitamin D binding protein and vitamin D receptor gene polymorphisms in Iranian patients with chronic periodontitis. Odontology. 2019;107(1):46-53. doi:10.1007/s10266-018-0383-0	Unrelated
14	Zuk AM, Quiñonez CR, Saarela O, Demmer RT, Rosella LC. Joint effects of serum vitamin D insufficiency and periodontitis on insulin resistance, pre-diabetes, and type 2 diabetes: results from the National Health and Nutrition Examination Survey (NHANES) 2009-2010. BMJ Open Diabetes Res Care. 2018;6(1):e000535. Published 2018 Jul 23. doi:10.1136/bmjdrc-2018-000535	No periodontitis group
15	Beyer K, Lie SA, Kjellevold M, Dahl L, Brun JG, Bolstad AI. Marine ω-3, vitamin D levels, disease outcome and periodontal status in rheumatoid arthritis outpatients. Nutrition. 2018;55-56:116-124. doi:10.1016/j.nut.2018.03.054	No periodontitis group
16	Yoshihara A, Kaneko N, Iwasaki M, Nohno K, Miyazaki H. Relationship between vitamin D receptor gene polymorphism and susceptibility to chronic kidney disease and periodontal disease in community-dwelling elderly. J Clin Periodontol. 2018;45(6):672-679. doi:10.1111/jcpe.12896	No periodontitis group
17	Dragonas P, Kaste LM, Nunn M, et al. Vitamin D deficiency and periodontal clinical attachment loss in HIV-seropositive women: A secondary analysis conducted in the Women's Interagency HIV Study (WIHS). Oral Surg Oral Med Oral Pathol Oral Radiol. 2018;125(6):567-573. doi:10.1016/j.oooo.2018.02.006	No periodontitis group
18	Luo PP, Xu HS, Chen YW, Wu SP. Periodontal disease severity is associated with micronutrient intake. Aust Dent J. 2018;63(2):193-201. doi:10.1111/adj.12606	Unsuitable control group
19	Khan FR, Ahmad T, Hussain R, Bhutta ZA. Relationship among Hypovitaminosis D, Maternal Periodontal Disease, and Low Birth Weight. J Coll Physicians Surg Pak. 2018;28(1):36-39. doi:10.29271/jcpsp.2018.01.36	Data presented in thresholds
20	Ho YP, Lin YC, Yang YH, et al. Association of vitamin D receptor gene polymorphisms and periodontitis in a Taiwanese Han population. J Dent Sci. 2017;12(4):360-367. doi:10.1016/j.jds.2017.07.001	Unrelated
21	Huang LG, Chen G, Chen DY, Chen HH. Factors associated with the risk of gingival disease in patients with rheumatoid arthritis. PLoS One. 2017;12(10):e0186346. Published 2017 Oct 12. doi:10.1371/journal.pone.0186346	No periodontitis group
22	Khan FR, Iqbal NT, Ahmed K, Ahmad T, Hussain R, Bhutta ZA. Effect of Vitamin D Supplementation on the Salivary Cytokines of Pregnant Women: A Randomized Placebo-Controlled Trial. J Int Acad Periodontol. 2017;19(4):118-125. Published 2017 Oct 1.	No periodontitis group
23	Tobón-Arroyave SI, Isaza-Guzmán DM, Pineda-Trujillo N. Association Study of Vitamin D Receptor (VDR) - Related Genetic Polymorphisms and their Haplotypes with Chronic Periodontitis in Colombian Population. J Clin Diagn Res. 2017;11(2):ZC60-ZC66. doi:10.7860/JCDR/2017/23967.9451	Unrelated
24	Romandini M, Gioco G, Perfetti G, Deli G, Staderini E, Laforì A. The association between periodontitis and sleep duration. J Clin Periodontol. 2017;44(5):490-501. doi:10.1111/jcpe.12713	No control group
25	Song W, Wang X, Tian Y, Zhang X, Lu R, Meng H. GC Gene Polymorphisms and Vitamin D-Binding Protein Levels Are Related	Unrelated

	to the Risk of Generalized Aggressive Periodontitis. Int J Endocrinol. 2016;2016:5141089. doi:10.1155/2016/5141089	
26	Heikkinen AM, Raivisto T, Kettunen K, et al. Pilot Study on the Genetic Background of an Active Matrix Metalloproteinase-8 Test in Finnish Adolescents. J Periodontol. 2017;88(5):464-472. doi:10.1902/jop.2016.160441	No periodontitis group
27	Baldodia A, Sharma RK, Tewari S, Narula SC. Effect of periodontitis on bone mineral density in postmenopausal women: A non-randomized interventional study. Quintessence Int. 2017;48(2):113-122. doi:10.3290/j.qi.a37132	Unable to access
28	Mangano F, Mortellaro C, Mangano N, Mangano C. Is Low Serum Vitamin D Associated with Early Dental Implant Failure? A Retrospective Evaluation on 1625 Implants Placed in 822 Patients. Mediators Inflamm. 2016;2016:5319718. doi:10.1155/2016/5319718	No periodontitis group
29	Woelber JP, Bremer K, Vach K, et al. An oral health optimized diet can reduce gingival and periodontal inflammation in humans - a randomized controlled pilot study [published correction appears in BMC Oral Health. 2016 Oct 6;16(1):109]. BMC Oral Health. 2016;17(1):28. Published 2016 Jul 26. doi:10.1186/s12903-016-0257-1	No supplementation
30	Khan FR, Ahmad T, Hussain R, Bhutta ZA. Vitamin D status and periodontal disease among pregnant and non-pregnant women in an underdeveloped district of Pakistan. J Int Soc Prev Community Dent. 2016;6(3):234-239. doi:10.4103/2231-0762.183111	Data presented in thresholds
31	Ferreira SM, Lima MH, Omena AL, et al. Prevalence of hypovitaminosis D and its association with oral lesions in HIV-infected Brazilian adults. Rev Soc Bras Med Trop. 2016;49(1):90-94. doi:10.1590/0037-8682-0159-2015	No control group
32	Pavlesen S, Mai X, Wactawski-Wende J, et al. Vitamin D Status and Tooth Loss in Postmenopausal Females: The Buffalo Osteoporosis and Periodontal Disease (OsteoPerio) Study. J Periodontol. 2016;87(8):852-863. doi:10.1902/jop.2016.150733	No periodontitis group
33	Eagle I, Benavides E, Eber R, et al. Periodontal health in breast cancer patients on aromatase inhibitors versus postmenopausal controls: a longitudinal analysis. J Clin Periodontol. 2016;43(8):659-667. doi:10.1111/jcpe.12562	No periodontal treatment
34	Bhavsar NV, Trivedi SR, Dulani K, Brahmbhatt N, Shah S, Chaudhri D. Clinical and radiographic evaluation of effect of risedronate 5 mg as an adjunct to treatment of chronic periodontitis in postmenopausal women (12-month study). Osteoporos Int. 2016;27(8):2611-2619. doi:10.1007/s00198-016-3577-8	Incomplete data
35	Penoni DC, Torres SR, Farias ML, Fernandes TM, Luiz RR, Leão AT. Association of osteoporosis and bone medication with the periodontal condition in elderly women. Osteoporos Int. 2016;27(5):1887-1896. doi:10.1007/s00198-015-3437-y	No periodontitis group
36	Wang X, Zhang TL, Chen D. Lack of association between the vitamin D receptor polymorphism rs2228570 and chronic periodontitis in a Han Chinese population. Genet Mol Res. 2015;14(4):12299-12305. Published 2015 Oct 9. doi:10.4238/2015.October.9.18	Unrelated
37	Dodington DW, Fritz PC, Sullivan PJ, Ward WE. Higher Intakes of Fruits and Vegetables, β-Carotene, Vitamin C, α-Tocopherol, EPA, and DHA Are Positively Associated with Periodontal Healing after Nonsurgical Periodontal Therapy in Nonsmokers but Not in Smokers. J Nutr. 2015;145(11):2512-2519. doi:10.3945/jn.115.211524	No supplementation

38	Lee HJ, Je DI, Won SJ, Paik DI, Bae KH. Association between vitamin D deficiency and periodontal status in current smokers. Community Dent Oral Epidemiol. 2015;43(5):471-478. doi:10.1111/cdoe.12173	No control group
39	Schulze-Späte U, Turner R, Wang Y, et al. Relationship of Bone Metabolism Biomarkers and Periodontal Disease: The Osteoporotic Fractures in Men (MrOS) Study. J Clin Endocrinol Metab. 2015;100(6):2425-2433. doi:10.1210/jc.2014-4180	Unrelated
40	Al-Mutairi KD, Al-Zahrani MS, Bahlas SM, Kayal RA, Zawawi KH. Periodontal findings in systemic lupus erythematosus patients and healthy controls. Saudi Med J. 2015;36(4):463-468. doi:10.15537/smj.2015.4.10746	No periodontitis group
41	El Jilani MM, Mohamed AA, Zeglam HB, et al. Association between vitamin D receptor gene polymorphisms and chronic periodontitis among Libyans. Libyan J Med. 2015;10:26771. Published 2015 Mar 19. doi:10.3402/ljm.v10.26771	Unrelated
42	Antonoglou GN, Suominen AL, Knuuttila M, et al. Associations between serum 25-hydroxyvitamin d and periodontal pocketing and gingival bleeding: results of a study in a non-smoking population in Finland. J Periodontol. 2015;86(6):755-765. doi:10.1902/jop.2015.140262	No periodontitis group
43	Amaliya A, Laine ML, Delanghe JR, Loos BG, Van Wijk AJ, Van der Velden U. Java project on periodontal diseases: periodontal bone loss in relation to environmental and systemic conditions. J Clin Periodontol. 2015;42(4):325-332. doi:10.1111/jcpe.12381	No periodontitis group
44	Antonenko O, Bryk G, Brito G, Pellegrini G, Zeni SN. Oral health in young women having a low calcium and vitamin D nutritional status. Clin Oral Investig. 2015;19(6):1199-1206. doi:10.1007/s00784-014-1343-x	No periodontitis group
45	Antonoglou GN, Knuuttila M, Niemelä O, et al. Low serum level of 1,25(OH)2 D is associated with chronic periodontitis. J Periodontal Res. 2015;50(2):274-280. doi:10.1111/jre.12207	Data previously published
46	Zhang X, Meng H, Xu L, et al. Vitamin d-binding protein levels in plasma and gingival crevicular fluid of patients with generalized aggressive periodontitis. Int J Endocrinol. 2014;2014:783575. doi:10.1155/2014/783575	Unrelated
47	Zhan Y, Samietz S, Holtfreter B, et al. Prospective Study of Serum 25-hydroxy Vitamin D and Tooth Loss. J Dent Res. 2014;93(7):639-644. doi:10.1177/0022034514534985	Unrelated
48	Zilahi E, Chen JQ, Papp G, Szántó A, Zeher M. Lack of association of vitamin D receptor gene polymorphisms/haplotypes in Sjögren's syndrome. Clin Rheumatol. 2015;34(2):247-253. doi:10.1007/s10067-014-2639-6	No periodontitis group
49	Millen AE, Andrews CA, LaMonte MJ, et al. Vitamin D status and 5-year changes in periodontal disease measures among postmenopausal women: the Buffalo OsteoPerio Study. J Periodontol. 2014;85(10):1321-1332. doi:10.1902/jop.2014.130686	No periodontitis group
50	Garcia MN. Vitamin D may reduce periodontal disease prevalence in older men. J Evid Based Dent Pract. 2014;14(1):39-41. doi:10.1016/j.jebdp.2014.01.010	No periodontitis group
51	Kaarthikeyan G, Jayakumar ND, Padmalatha O, Varghese S, Anand B. Analysis of association of TaqI VDR gene polymorphism with the chronic periodontitis in Dravidian ethnicity. Indian J Hum Genet. 2013;19(4):465-468. doi:10.4103/0971-6866.124377	Unrelated

52	Sahli MW, Wactawski-Wende J, Ram PK, et al. Association of plasma 25-hydroxyvitamin d concentrations and pathogenic oral bacteria in postmenopausal females. J Periodontol. 2014;85(7):944-955. doi:10.1902/jop.2013.130518	No periodontitis group
53	Karasneh JA, Ababneh KT, Taha AH, et al. Association of vitamin D receptor gene polymorphisms with chronic and aggressive periodontitis in Jordanian patients. Eur J Oral Sci. 2013;121(6):551-558. doi:10.1111/eos.12085	Unrelated
54	Baldini A, Nota A, Fanti E, Martelli FS, Ottomano C, Lippi G. Association between periodontal disease and Interleukin-1β +3953 and vitamin D receptor Taq1 genetic polymorphisms in an Italian caucasian population. Ann Stomatol (Roma). 2013;4(2):191-195. Published 2013 Jun 25.	Unrelated
55	Antonoglou G, Knuuttila M, Niemelä O, et al. Serum 1,25(OH)D level increases after elimination of periodontal inflammation in T1DM subjects. J Clin Endocrinol Metab. 2013;98(10):3999-4005. doi:10.1210/jc.2013-1906	Unsuitable control group
56	Scapoli L, Girardi A, Palmieri A, et al. IL6 and IL10 are genetic susceptibility factors of periodontal disease. Dent Res J (Isfahan). 2012;9(Suppl 2):S197-S201. doi:10.4103/1735-3327.109754	Unrelated
57	Alshouibi EN, Kaye EK, Cabral HJ, Leone CW, Garcia RI. Vitamin D and periodontal health in older men. J Dent Res. 2013;92(8):689-693. doi:10.1177/0022034513495239	No periodontitis group
58	Bastos Jdo A, Andrade LC, Ferreira AP, et al. Serum levels of vitamin D and chronic periodontitis in patients with chronic kidney disease. J Bras Nefrol. 2013;35(1):20-26. doi:10.5935/01012800.20130004	Non healthy patients
59	Johnston BD, Fritz PC, Ward WE. Use of dietary supplements in patients seeking treatment at a periodontal clinic. Nutrients. 2013;5(4):1110-1121. Published 2013 Apr 2. doi:10.3390/nu5041110	No periodontitis group
60	Hiremath VP, Rao CB, Naik V, Prasad KV. Anti-inflammatory effect of vitamin D on gingivitis: a dose-response randomised control trial. Oral Health Prev Dent. 2013;11(1):61-69. doi:10.3290/j.ohpd.a29377	Unrelated
61	Jimenez M, Giovannucci E, Krall Kaye E, Joshipura KJ, Dietrich T. Predicted vitamin D status and incidence of tooth loss and periodontitis. Public Health Nutr. 2014;17(4):844-852. doi:10.1017/S1368980013000177	No periodontitis group
62	Millen AE, Hovey KM, LaMonte MJ, et al. Plasma 25-hydroxyvitamin D concentrations and periodontal disease in postmenopausal women. J Periodontol. 2013;84(9):1243-1256. doi:10.1902/jop.2012.120445	No periodontitis group
63	Yoshihara A, Iwasaki M, Miyazaki H, Nakamura K. Association between low renal function and tooth loss over 5 years. Gerodontology. 2014;31(2):111-116. doi:10.1111/ger.12015	No periodontitis group
64	Adegboye AR, Christensen LB, Holm-Pedersen P, Avlund K, Boucher BJ, Heitmann BL. Intake of dairy products in relation to periodontitis in older Danish adults. Nutrients. 2012;4(9):1219-1229. doi:10.3390/nu4091219	Abstract
65	Wang Y, Sugita N, Yoshihara A, et al. Peroxisome proliferator-activated receptor (PPAR) γ polymorphism, vitamin D, bone mineral density and periodontitis in postmenopausal women. Oral Dis. 2013;19(5):501-506. doi:10.1111/odi.12032	No periodontitis group

66	Jönsson D, Aggarwal P, Nilsson BO, Demmer RT. Beneficial effects of hormone replacement therapy on periodontitis are vitamin D associated. J Periodontol. 2013;84(8):1048-1057. doi:10.1902/jop.2012.120434	No periodontitis group
67	Rivas-Tumanyan S, Spiegelman D, Curhan GC, Forman JP, Joshipura KJ. Periodontal disease and incidence of hypertension in the health professionals follow-up study. Am J Hypertens. 2012;25(7):770-776. doi:10.1038/ajh.2012.32	No periodontitis group
68	Zhou X, Han J, Song Y, Zhang J, Wang Z. Serum levels of 25-hydroxyvitamin D, oral health and chronic obstructive pulmonary disease. J Clin Periodontol. 2012;39(4):350-356. doi:10.1111/j.1600-051X.2012.01852.x	No periodontitis group
69	Teles FR, Teles RP, Martin L, Socransky SS, Haffajee AD. Relationships among interleukin-6, tumor necrosis factor-α, adipokines, vitamin D, and chronic periodontitis. J Periodontol. 2012;83(9):1183-1191. doi:10.1902/jop.2011.110346	No control group
70	Martelli FS, Mengoni A, Martelli M, Rosati C, Fanti E. VDR TaqI polymorphism is associated with chronic periodontitis in Italian population. Arch Oral Biol. 2011;56(12):1494-1498. doi:10.1016/j.archoralbio.2011.06.012	Unrelated
71	Bashutski JD, Eber RM, Kinney JS, et al. The impact of vitamin D status on periodontal surgery outcomes. J Dent Res. 2011;90(8):1007-1012. doi:10.1177/0022034511407771	Surgical periodontal treatment
72	Mesa F, Gonzalez A, Souki N, et al. Alveolar bone level is not associated with vitamin D receptor gene polymorphism and bone density in mandible. Clin Oral Investig. 2012;16(2):371-377. doi:10.1007/s00784-011-0536-9	Unrelated
73	Bashutski JD, Eber RM, Kinney JS, et al. Teriparatide and osseous regeneration in the oral cavity. N Engl J Med. 2010;363(25):2396-2405. doi:10.1056/NEJMoa1005361	Surgical periodontal treatment
74	Garcia MN, Hildebolt CF, Miley DD, et al. One-year effects of vitamin D and calcium supplementation on chronic periodontitis. J Periodontol. 2011;82(1):25-32. doi:10.1902/jop.2010.100207	No periodontal treatment
75	Boggess KA, Espinola JA, Moss K, Beck J, Offenbacher S, Camargo CA Jr. Vitamin D status and periodontal disease among pregnant women. J Periodontol. 2011;82(2):195-200. doi:10.1902/jop.2010.100384	Pregnant women
76	Jabbar S, Drury J, Fordham J, Datta HK, Francis RM, Tuck SP. Plasma vitamin D and cytokines in periodontal disease and postmenopausal osteoporosis. J Periodontal Res. 2011;46(1):97-104. doi:10.1111/j.1600-0765.2010.01317.x	Unrelated
77	Liu K, Meng H, Lu R, et al. Initial periodontal therapy reduced systemic and local 25-hydroxy vitamin D(3) and interleukin-1beta in patients with aggressive periodontitis. J Periodontol. 2010;81(2):260-266. doi:10.1902/jop.2009.090355	No supplementation
78	Kobayashi T, Nagata T, Murakami S, et al. Genetic risk factors for periodontitis in a Japanese population. J Dent Res. 2009;88(12):1137-1141. doi:10.1177/0022034509350037	Unrelated
79	Miley DD, Garcia MN, Hildebolt CF, et al. Cross-sectional study of vitamin D and calcium supplementation effects on chronic periodontitis. J Periodontol. 2009;80(9):1433-1439. doi:10.1902/jop.2009.090077	No periodontal treatment
80	Kim TW, Alford DP, Holick MF, Malabanan AO, Samet JH. Low vitamin d status of patients in methadone maintenance treatment. J	No periodontitis group

	Addict Med. 2009;3(3):134-138. doi:10.1097/ADM.0b013e31819b736d	
81	Borges MA, Figueiredo LC, Brito RB Jr, Faveri M, Feres M. Microbiological composition associated with vitamin D receptor gene polymorphism in chronic periodontitis. Braz Oral Res. 2009;23(2):203-208. doi:10.1590/s1806-83242009000200018	Unrelated
82	Liu K, Meng H, Tang X, et al. Elevated plasma calcifediol is associated with aggressive periodontitis. J Periodontol. 2009;80(7):1114-1120. doi:10.1902/jop.2009.080675	Unrelated
83	Dixon D, Hildebolt CF, Miley DD, et al. Calcium and vitamin D use among adults in periodontal disease maintenance programmes. Br Dent J. 2009;206(12):627-617. doi:10.1038/sj.bdj.2009.519	Unrelated
84	Wu Y, Shu R, Luo LJ, Ge LH, Xie YF. Initial comparison of proteomic profiles of whole unstimulated saliva obtained from generalized aggressive periodontitis patients and healthy control subjects. J Periodontal Res. 2009;44(5):636-644. doi:10.1111/j.1600-0765.2008.01172.x	Unrelated
85	Wang C, Zhao H, Xiao L, et al. Association between vitamin D receptor gene polymorphisms and severe chronic periodontitis in a Chinese population. J Periodontol. 2009;80(4):603-608. doi:10.1902/jop.2009.080465	Unrelated
86	Alvim-Pereira F, Montes CC, Thomé G, Olandoski M, Trevilatto PC. Analysis of association of clinical aspects and vitamin D receptor gene polymorphism with dental implant loss. Clin Oral Implants Res. 2008;19(8):786-795. doi:10.1111/j.1600-0501.2008.01532.x	Unrelated
87	Buduneli N, Saygan BH, Karaduman U, Saraç F, Karaduman M, Ayçelik N. Calcium, vitamin D supplements with or without alendronate and supragingival calculus formation in osteoporotic women: a preliminary study. Expert Opin Pharmacother. 2008;9(12):2015-2020. doi:10.1517/14656566.9.12.2015	Unrelated
88	Nibali L, Parkar M, D'Aiuto F, et al. Vitamin D receptor polymorphism (-1056 Taq-I) interacts with smoking for the presence and progression of periodontitis. J Clin Periodontol. 2008;35(7):561-567. doi:10.1111/j.1600-051X.2008.01233.x	Unrelated
89	Gunes S, Sumer AP, Keles GC, et al. Analysis of vitamin D receptor gene polymorphisms in patients with chronic periodontitis. Indian J Med Res. 2008;127(1):58-64.	Unrelated
90	Li S, Yang MH, Zeng CA, et al. Association of vitamin D receptor gene polymorphisms in Chinese patients with generalized aggressive periodontitis. J Periodontal Res. 2008;43(3):360-363. doi:10.1111/j.1600-0765.2007.01044.x	Unrelated
91	de Souza CM, Braosi AP, Luczyszyn SM, et al. Association between vitamin D receptor gene polymorphisms and susceptibility to chronic kidney disease and periodontitis. Blood Purif. 2007;25(5-6):411-419. doi:10.1159/000109235	Unrelated
92	Naito M, Miyaki K, Naito T, et al. Association between vitamin D receptor gene haplotypes and chronic periodontitis among Japanese men. Int J Med Sci. 2007;4(4):216-222. Published 2007 Aug 22. doi:10.7150/ijms.4.216	Unrelated
93	Nibali L, Ready DR, Parkar M, et al. Gene polymorphisms and the prevalence of key periodontal pathogens. J Dent Res. 2007;86(5):416-420. doi:10.1177/154405910708600505	Unrelated
94	Park KS, Nam JH, Choi J. The short vitamin D receptor is associated with increased risk for generalized aggressive periodontitis. J Clin	Unrelated

	Periodontol. 2006;33(8):524-528. doi:10.1111/j.1600-051X.2006.00944.x	
95	Brett PM, Zygogianni P, Griffiths GS, et al. Functional gene polymorphisms in aggressive and chronic periodontitis. J Dent Res. 2005;84(12):1149-1153. doi:10.1177/154405910508401211	Unrelated
96	Wactawski-Wende J, Hausmann E, Hovey K, Trevisan M, Grossi S, Genco RJ. The Association Between Osteoporosis and Alveolar Crestal Height in Postmenopausal Women. J Periodontol. 2005;76 Suppl 11S:2116-2124. doi:10.1902/jop.2005.76.11-S.2116	Unrelated
97	Dietrich T, Nunn M, Dawson-Hughes B, Bischoff-Ferrari HA. Association between serum concentrations of 25-hydroxyvitamin D and gingival inflammation. Am J Clin Nutr. 2005;82(3):575-580. doi:10.1093/ajcn.82.3.575	No periodontitis group
98	de Brito Júnior RB, Scarel-Caminaga RM, Trevilatto PC, de Souza AP, Barros SP. Polymorphisms in the vitamin D receptor gene are associated with periodontal disease. J Periodontol. 2004;75(8):1090-1095. doi:10.1902/jop.2004.75.8.1090	Unrelated
99	Dietrich T, Joshipura KJ, Dawson-Hughes B, Bischoff-Ferrari HA. Association between serum concentrations of 25-hydroxyvitamin D3 and periodontal disease in the US population. Am J Clin Nutr. 2004;80(1):108-113. doi:10.1093/ajcn/80.1.108	No periodontitis group
100	Inagaki K, Krall EA, Fleet JC, Garcia RI. Vitamin D receptor alleles, periodontal disease progression, and tooth loss in the VA dental longitudinal study. J Periodontol. 2003;74(2):161-167. doi:10.1902/jop.2003.74.2.161	No periodontitis group
101	Sun JL, Meng HX, Cao CF, et al. Relationship between vitamin D receptor gene polymorphism and periodontitis. J Periodontal Res. 2002;37(4):263-267. doi:10.1034/j.1600-0765.2002.01605.x	No periodontitis group
102	Yoshihara A, Sugita N, Yamamoto K, Kobayashi T, Miyazaki H, Yoshi H. Analysis of vitamin D and Fcgamma receptor polymorphisms in Japanese patients with generalized early-onset periodontitis. J Dent Res. 2001;80(12):2051-2054. doi:10.1177/00220345010800120501	Unrelated
103	Krall EA, Wehler C, Garcia RI, Harris SS, Dawson-Hughes B. Calcium and vitamin D supplements reduce tooth loss in the elderly. Am J Med. 2001;111(6):452-456. doi:10.1016/s0002-9343(01)00899-3	No periodontal treatment
104	Murthykumar K, Arjunkumar R, Jayaseelan VP. Association of vitamin D receptor gene polymorphism (rs10735810) and chronic periodontitis. J Investig Clin Dent. 2019;10(4):e12440. doi:10.1111/jicd.12440	No periodontitis group
105	Krall EA, Garcia RI, Dawson-Hughes B. Increased risk of tooth loss is related to bone loss at the whole body, hip, and spine. Calcif Tissue Int. 1996;59(6):433-437. doi:10.1007/BF00369206	Unrelated
106	Krayer JW, Emerson DL, Goldschmidt-Clermont PJ, Nel AE, Werner PA, Galbraith RM. Qualitative and quantitative studies of Gc (vitamin D-binding protein) in normal subjects and patients with periodontal disease. J Periodontal Res. 1987;22(4):259-263. doi:10.1111/j.1600-0765.1987.tb01583.x	Unrelated
107	Spiller WF Jr. A clinical evaluation of calcium therapy for periodontal disease. Dent Dig. 1971;77(9):522-526.	Unable to access
108	Cheraskin E, Ringsdorf WM Jr. Effect of regular versus sustained-release multivitamin supplementation upon periodontal parameters. Gingival state. Int Z Vitaminforsch. 1969;39(3):314-319.	Unable to access

109	Cheraskin E, Ringsdorf WM Jr. Effect of regular versus sustained-release multivitamin supplementation upon periodontal parameters. II. Sulcus depth and clinical tooth mobility. Int Z Vitaminforsch. 1969;39(4):476-485.	Unable to access

Appendix S3. Newcastle-Ottawa Scale

Study	SELECTION				COMPARABILITY	EXPOSURE			RoB Score
	Is the case definition adequate?	Representativeness of the cases?	Selection of controls?	Definition of controls?	Comparability of cases and controls of design or analysis?	Ascertainment of exposure?	Same method of ascertainment for cases and controls	Non-response rate?	
Isola et al. 2020	a	a	a	a	a/b	a	a	a	9
Costantini et al. 2020	a	b	a	a	a/b	a	a	a	8
Agrawal et al. 2019	a	b	a	a	a/b	a	a	a	8
Ketharanathan et al. 2019	a	b	c	a	a	a	a	a	6
Ebersole et al. 2018	a	a	a	a	a/b	a	a	a	9
Anbarcioglu et al. 2018	a	a	a	a	a/b	a	a	a	9
Balci Yuce et al. 2017	a	b	a	a	a/b	a	a	a	8
Laky et al. 2017	a	a	a	a	a/b	a	a	a	9
Abreu et al. 2016	a	b	c	a	a/b	a	a	a	7
Joseph et al. 2015	a	a	b	a	a/b	a	a	a	8
Antonoglou et al. 2015	a	b	c	a	a/b	a	a	a	7
Miricescu et al. 2014	a	b	c	a	a/b	a	a	a	7
Zhang et al. 2012	a	b	c	a	a/b	a	a	a	7

Appendix S4. RoB2 Tool

	R	D	Mi	Me	S	O
Gao et al. 2020	+	+	+	+	+	+

R - Bias arising from the randomisation process; **D -** Bias due to deviations from intended interventions; **Mi -** Bias due to missing outcome data; **Me -** Bias in measurement of the outcome; **S -** Bias in selection of the reported result; **O -** Overall risk of bias.

Appendix S5. ROBINS-I Tool

Domain	1	2	3	4	5	6	7	
Study	Confounding	Selection	Classification	Deviations from interventions	Missing Data	Measurement of Outcomes	Selection of Reported Result	Overall
Perayil et al. (2015)	Low	Low	Low	Low	Low	Low	Low	Low

Appendix S6. Summary of estimates of meta-regression to assess the influence of smoking on 25(OH)D serum levels

Variable	N	Estimate	SE	95% CI	*p-value*
Smoking habits	10	0.0671	0.17	-0.27 - 0.40	0.400
